# Gleanings from Our Exchanges

**Published:** 1873-06-01

**Authors:** 


Horehound in Coughs of Children.—Horehound, the herb of *Marrubrium Vulgare*, is an old remedy, and it is doubtful if we have any other that surpasses it as a remedy for the winter and spring coughs children are so much subject to. It has a decided soothing influence on the laryngeal and pneumogastric nerves. A strong decoction of the herb, sweetened with honey, makes an excellent cough remedy which children seldom refuse, and it cures their coughs without much delay. — *St. Louis Medical Archives.*

Digitalis in the Dropsies and Nephritic Diseases of Children.— Dr. Cooper, of Indianapolis, claims for digitalis a specific action in curing the dropsies and other nephritic complications, arising as a sequel from scarlatina. He gives several cases where ascites, anasarca, albuminuria and emaciation rapidly disappeared immediately after the patient was put on small doses of tinct. of digitalis; without any other treatment the cases recovered perfectly.— *St. Louis Medical Archives.*

Gelsemium in Gynæcology.— Dr. H. V. Ferrell, Carbondale, Ill., writes to the *Clinic*, that two doses of gelsemium, of ten drops each, entirely relieved the excessive irritability of the uterus in a case of endometritis, and the os, which was, before the admistration of gelsemium, rigid and contracted, dilated. Another case — suppression of lochia, and retention of part of the placenta — the result of the stupidity of a midwife. "Present condition of the patient alarming; in which, on examination, the os was found firmly contracted, and hardly admitted the end of the index finger. The patient was treated with gelsemium and belladonna, and in twelve hours the os dilated so as to admit two fingers, and permit the extraction of the retained placenta, etc., when the patient recovered without further trouble.

Gelsemium (in five drops every hour, in water, till relief is obtained, and then at longer intervals) has relieved after-pains in very severe cases. Dr. Shaw, of Guthrie, Iowa, claims to have had the best results from gelsemium in the treatment of uterine leucorrhœa, and Dr. Mobsworth, of DesMoines, places more reliance in it for the successful treatment of chronic dysmenorrhœa than in any other remedy. Both these gentlemen say its good effects in these affections are frequently as prompt and decided as is quinia in intermittents.— *St. Louis Medical Archives.*

Morphia as a Parturient.— The *Pacific Medical and Surgical Journal* comments on a case reported in the *Clinic* by Dr. Urmoston, of Ill., in which a dose of morphia administered for the purpose of checking what he supposed to be "false pains" really induced a rapid dilation of the os, and a speedy delivery. Dr. Urmoston attributed the promptness of the morphia in this case to its effects in removing a rheumatic state of the uterus. The *Pacific Journal* comments in the following language. "In a slow and painful labor, with little disposition to relaxation of the os uteri, a large dose of morphia will almost invariably prove an active parturifacient. It relieves the pain, and while it appears to arrest labor, relaxation takes place, and the child steals a march on the accoucheur.

